# Cellular Senescence Affects Cardiac Regeneration and Repair in Ischemic Heart Disease

**DOI:** 10.14336/AD.2020.0811

**Published:** 2021-04-01

**Authors:** Chi Yan, Zhimeng Xu, Weiqiang Huang

**Affiliations:** ^1^Department of Geriatric Cardiology, The First Affiliated Hospital of Guangxi Medical University, Guangxi, China.; ^2^Guangxi Key Laboratory of Precision Medicine in Cardio-cerebrovascular Diseases Control and Prevention, Guangxi, China.; ^3^Department of Cardiology, Guangxi Clinical Research Center for Cardio-cerebrovascular Diseases, Guangxi, China.; ^4^Department of Cardiology, The People's Hospital of Guangxi Zhuang Autonomous Region, Guangxi, China.

**Keywords:** senescence, ischemic heart disease (IHD), angiogenesis, myogenesis

## Abstract

Ischemic heart disease (IHD) is defined as a syndrome of ischemic cardiomyopathy. Myogenesis and angiogenesis in the ischemic myocardium are important for cardiomyocyte (CM) survival, improving cardiac function and decreasing the progression of heart failure after IHD. Cellular senescence is a state of permanent irreversible cell cycle arrest caused by stress that results in a decline in cellular functions, such as proliferation, migration, homing, and differentiation. In addition, senescent cells produce the senescence-associated secretory phenotype (SASP), which affects the tissue microenvironment and surrounding cells by secreting proinflammatory cytokines, chemokines, growth factors, and extracellular matrix degradation proteins. The accumulation of cardiovascular-related senescent cells, including vascular endothelial cells (VECs), vascular smooth muscle cells (VSMCs), CMs and progenitor cells, is an important risk factor of cardiovascular diseases, such as vascular aging, atherosclerotic plaque formation, myocardial infarction (MI) and ventricular remodeling. This review summarizes the processes of angiogenesis, myogenesis and cellular senescence after IHD. In addition, this review focuses on the relationship between cellular senescence and cardiovascular disease and the mechanism of cellular senescence. Finally, we discuss a potential therapeutic strategy for MI targeting senescent cells.

## Introduction

Globally, cardiovascular disease is the leading cause of death among noncommunicable diseases, and ischemic heart disease (IHD) is the number one cause of cardiovascular disease-related death[1]. IHD is defined as a syndrome of ischemic cardiomyopathy, and patients experience acute myocardial infarction (AMI) and subsequent ventricular dysfunction until progressive heart failure develops [2]. The degree of myocardial injury and cardiomyocyte (CM) death depends on the size of the infarct area, the duration of ischemia and the efficiency of reperfusion after MI (myocardial infarction), and the loss of a large number of CMs subsequently causes pathological remodeling and heart failure [3, 4]. Currently, the main treatment for myocardial infarction is revascularization, which can be achieved by coronary artery intervention and coronary artery bypass grafting; however, approximately 1/5 of patients with coronary heart disease cannot undergo revascularization by these two methods [5, 6]. Numerous studies have demonstrated that inducing CM proliferation and promoting cardiac regeneration are promising methods for treating MI [7]. One strategy for myogenesis is exploiting the limited regenerative capacity of CMs by reactivating the proliferation of existing CMs through dedifferentiation, reentry into the cell cycle, and cytokinesis[8]. In addition, cardiac stem cells (CSCs) can differentiated to new CMs, and treatments based on CSCs represent a promising emerging approach to replace lost tissue and restore cardiac contractility [9]. Moreover, another therapeutic strategy called therapeutic angiogenesis could be used to enhance the formation of new blood vessels or maturation of the pre-existing vasculature to bypass the occlusive arteries and recover organ perfusion [10]. Notably, cardiac regeneration after MI is driven by new CM formation and requires a supportive vascular network [11]. Additionally, angiogenesis formed of new blood vessels during the late phases of remodeling provides an oxygen supply for efficient myocardial reconstruction [12, 13]. At the cellular level, the processes of myogenesis and angiogenesis require the participation of endothelial cells (ECs), vascular smooth muscle cells (VSMCs), CMs, and some stem cells.

Aging is a process of degeneration at the organismal, cellular, and molecular levels, and senescence is one of the causative processes of aging and is responsible for aging-related disorders [14]. Cellular senescence is permanent and irreversible cell cycle arrest caused by different stresses and is insensitive to growth factor and mitogen stimulation [15]. Senescence cells secrete some inflammatory signaling factors, including interleukins, inflammatory cytokines, growth factors, secreted proteases, and extracellular matrix proteins were termed the SASP [16, 17]. Cellular senescence is not only a key driver of atherosclerosis but also an independent risk factor for MI and an important cause of high mortality after MI[18, 19]. Senescence in CMs, vascular cells and stem/progenitor cells entails a decreased replicative capacity and cellular dysfunction, which contribute to cardiovascular aging and impairment of the reparative and regenerative potential [20, 21].This review summarizes the mechanisms of cellular senescence and the SASP and the effects of cellular senescence on the process of IHD to illuminate potential therapies targeting senescent cells.

### Myogenesis after MI

The hearts of newborn mammals maintain a transient ability to regenerate at birth, and this ability is gradually lost. In healthy, uninjured adults, the CM turnover rate is approximately 0.5% to 2% per year [22]. After MI, CM necrosis in the infarcted area and cellular dysfunction in the peri-infarcted area are the primary causes of ventricular remodeling and heart failure [23, 24]. CM renewal is slow, and many CMs are lost after MI, leading to the infarcted myocardium being replaced by scar tissue formed from the extracellular matrix [23, 24]. However, the scar tissue that replaces the myocardium lacks the structure and function of myocardial cells and cannot perform normal myocardial contractile activities; thus, myocardial regeneration plays a vital role in repairing myocardial tissue and improving the prognosis of MI patients [25]. Currently, research investigating endogenous CM regeneration mainly involves the proliferation of original CMs and the differentiation of myocardial stem/progenitor cells or other stem cells into CMs [26, 27]. Studies have indicated that CSCs have the abilities of self-renewal, cloning and differentiation into CMs, smooth muscle cells and VECs [28]. MSCs can promote cardiac regeneration by activating CSCs or increasing the viability of nearby CMs by paracrine growth factors and recruiting stem cells from the bone marrow to increase angiogenesis [29]. Therefore, identifying an approach to increase CM proliferation is necessary; for instance, certain signaling pathways and secreted factors can lead CMs to dedifferentiate and re-enter the cell cycle [27, 30].

### Angiogenesis after MI

MI occurs because a reduction or interruption in the coronary blood flow leads to myocardial death [31]. Studies have shown that enhanced angiogenesis can improve myocardial cell survival proximal to the infarct area and inhibit ventricular remodeling of the infarcted heart [31]. Angiogenesis refers to the formation of new capillaries by sprouting or splitting of preexisting vessels [5]. On the one hand, vascular endothelial cells (VECs) are a basic component of blood vessels, and their proliferation is essential for the formation of new blood vessels [32]. VSMCs are essential for maintaining vascular wall elasticity and vascular movement, while inflammation changes the phenotype of VSMCs, promoting the formation of atherosclerosis and accelerating MI [33]. On the other hand, angiogenesis requires vascular stem/progenitor cells, which mainly include mesenchymal stem cells (MSCs), smooth muscle progenitor cells (SMPCs), endothelial progenitor cells (EPCs) and pericytes, which are derived from bone marrow, circulating peripheral blood, blood vessel walls or other extravascular tissues; these cells are acquired through proliferation, differentiation, homing and secretion and participate in the repair of damaged blood vessels and angiogenesis [34-37]. An increasing number of studies have shown that the targeted regulation of angiogenesis is conducive to vascular repair and prevent the development of diseases. For example, MSCs can secrete angiogenesis-related proteins or exosomes and promote angiogenesis through paracrine effects [38]. Anderson JD et al found that MSC-derived exosomes can regulate ECs and promote angiogenesis through the NF-KB signaling pathway [39]. In addition, EPCs overexpressing miR-205 can significantly enhance vascular regeneration in vivo and in vitro [40]. The overexpression of miR-126-3p increases EPC migration, promotes luminal structure formation, and increases the capillary density [41].

### Hallmarks of cellular senescence

**Figure 1.**
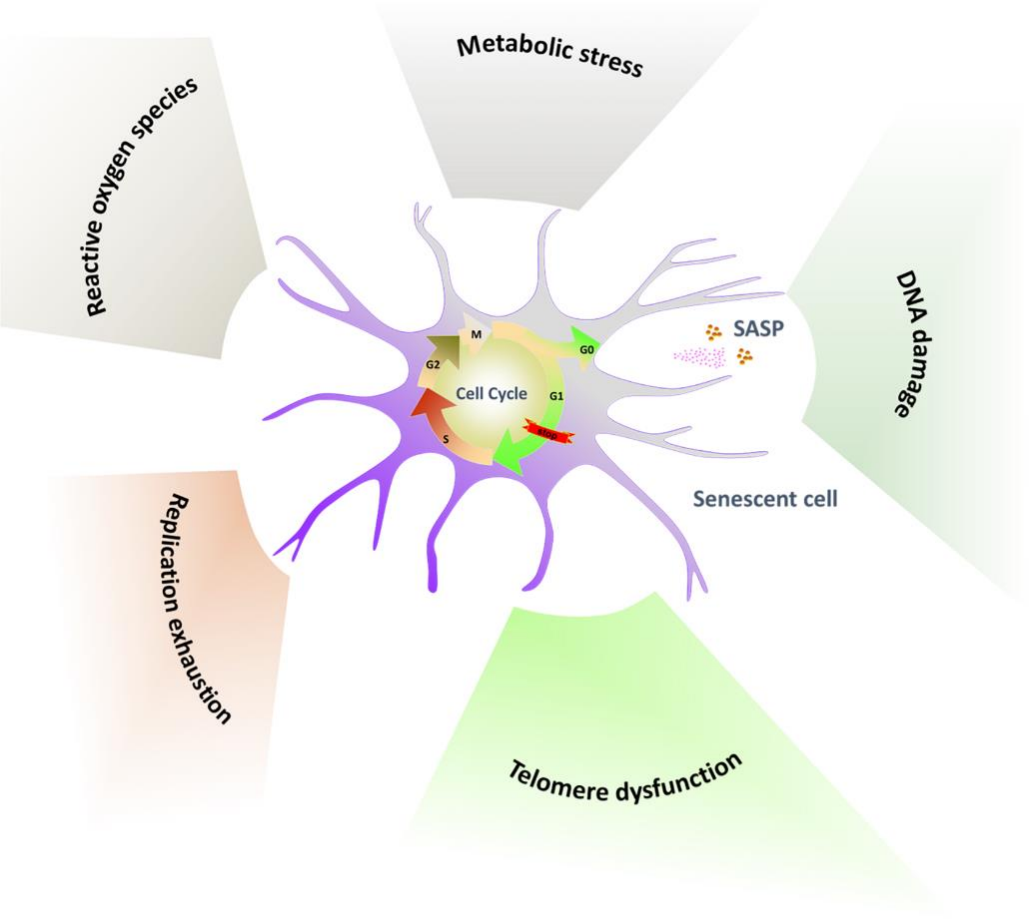
Hallmarks of cellular senescence. Reactive oxygen species, replication exhaustion, telomere dysfunction, DNA damage and active oncogenes, metabolic stress primarily drive stable cell cycle arrest. Exposure to different types of stresses drive cellular senescence, and cellular senescence in turn secret the SASP.

Cellular senescence was identified as a stable cell cycle arrest induced by damage or stress applied to proliferating cells [42]. Besides, the SASP is the most relevant phenotypic program implemented in senescent cells [43] (Fig. 1). Senescent cells exhibit an enlarged, irregular morphology and increase with senescence-associated β-galactosidase (SA-β-Gal), which is a hydrolase found in lysosomes [44]. The primary drivers of cellular senescence include reactive oxygen species, replication exhaustion, telomere dysfunction, DNA damage and active oncogenes, metabolic stress [42, 45]. Exposure to different types of acute sub-lethal stresses, such as oxidative stress and DNA damaging agents, was shown to induce cellular senescence in different cell types within relatively short periods [46], while replication exhaustion describes a senescent state with telomere shortening or dysfunctional telomeres after serial cultivation [47]. In 1961, Hayflick *et al*. first cultured fibroblasts from embryos and adults in vitro and found that the cells could not divide indefinitely, even under optimal cell culture, because of the change in their internal factors [48]. The main internal factor hindering cell replication was subsequently identified as telomere shortening [49]. Telomeres are nucleoprotein complexes responsible for the genomic integrity of chromosome ends and have a repetitive nucleotide sequence 5'-TTAGGG-3' at the end of chromosomes in humans, which is essential for ensuring chromosome stability [50, 51]. Over time, telomeres are shortened with the repetitive cycles because of the end-replication problem, and telomere attrition is regarded as a marker of cellular senescence [51, 52]. The SASP may have a major effect on the physiology of old organisms and can be responsible for chronic inflammation and age-linked diseases [17]. Importantly, the SASP reinforces the senescence growth arrest in vitro by implementing an autocrine positive-feedback loop and induces neighboring cells to undergo senescence [53].

## Molecular mechanisms of cellular senescence and SASP

### Pathways regulating the cell cycle

**Figure 2.**
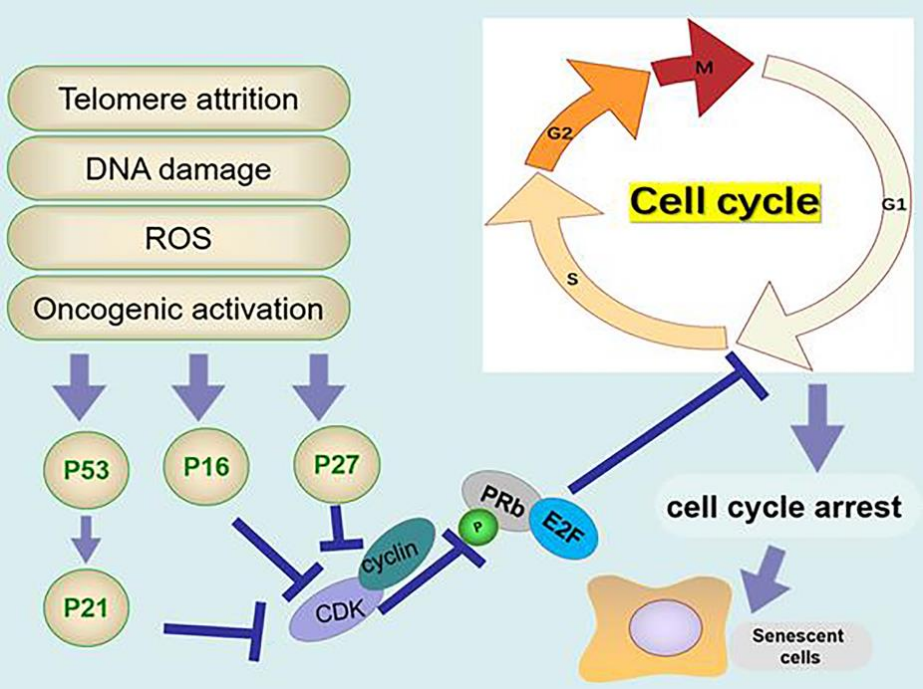
The molecular mechanism of cellular senescence. Telomere attrition, DNA damage, reactive oxygen species (ROS) and oncogenic activation activate the expression of p16, P53/P21 and P27. P16, P53/P21 and P27 inhibit the phosphorylation of retinoblastoma protein (Rb) and inhibit the transcription of E2F, resulting in cell cycle arrest.

In response to various intrinsic and extrinsic stresses, cells can engage the tumor suppressor pathways P53-P21, P16-RB and/or P27 effector pathways to arrest the cell cycle in an attempt to mitigate the damage that occurred (Fig. 2).

p53 is a common tumor suppressor that controls the transcription of extensive gene networks involved in apoptosis, cell cycle arrest, DNA damage repair, and aging[54]. Physiological p53 activity can prevent cancer and aging, while unrestricted and excessive p53 activity can promote aging [5]. P53 is activated by DNA damage, hypoxia, and oxidative stress [55]. Telomere damage is a type of double-strand DNA damage that triggers a DDR signal and then activates P53 to upregulate the CDKN1A-encoded cyclin-dependent kinase inhibitor p21 [56]. The transcription factor E2F plays a key role in regulating the cell cycle in the cell cycle G1-S phase [55]. An increase in P21 can inhibit the activity of cyclin E/A/Cdk2 and the phosphorylation of retinoblastoma protein (Rb); nonphosphorylated Rb inhibits its transcriptional activity by binding E2F and prevents the expression of cell cycle genes, resulting in cell cycle phase arrest [55, 57-60].

P16 is a marker of senescent cells and a CDK inhibitor [61]. It has been shown that P16INK4a can increase during tissue injury, inflammation, tumorigenesis and aging. Downregulating the expression of P16^INK4a^ weakens the SASP and improves the healthy life span of physiologically aging mice [62].The activation of the CDKN2A gene produces p16^INK4A^, which directly inhibits cyclin D/CDK4/6, Rb phosphorylation and E2F transcriptional activity [63, 64].

P27^kip1^ is a CDK inhibitor that regulates the cell cycle, apoptosis and cell movement [65]. P27^kip1^ is a key inhibitor in the G1/S phase of mammalian cells, the overexpression of p27^kip1^ increases the expression of P27^kip1^, and critically promotes cell growth arrest in replicative senescence [66]. Promoting p27^Kip1^ubiquitin-proteasome degradation by SIRT6 can delay cellular senescence [67].

### Senescence- and SASP-related signaling pathways

Mammalian TOR (MTOR) is a serine/threonine-specific protein kinase that plays a central regulatory role in chronic stress, mitochondrial dysfunction and autophagy associated with senescence [68, 69]. MTOR is a negative regulator of autophagy, and autophagy plays a vital role in maintaining physiological function and adapting to various cellular stresses by degrading organelles and misfolded proteins [70]. Inadequate autophagy leads to the accumulation of oxidative damage, loss of proteostasis, genomic instability and epigenomic alteration, which trigger cellular senescence and cause cardiac aging [71, 72]. Sung JY et al found that activating the mTOR pathway can induce VSMC senescence by reducing the levels of signal-associated autophagy proteins [73]. Laberge RM et al showed that mTOR drives the SASP and that suppressing mTOR complex1 can repress the transcriptional activity of NF-κB leading to the downregulation of SASP factors such as IL-6, IL-8, monocytes, chemotactic proteins [74].

GATA-binding factor 4 (GATA4) belongs to the family of GATA transcription factors, which are regulators of cellular senescence and the SASP [75, 76]. Abnormal GATA4 expression can induce senescence, while interfering with GATA4 expression can inhibit senescence [75]. GATA4 is degraded via selective autophagy [75]. Mutant ataxia telangiectasia mutation (ATM) and ataxia telangiectasia and Rad3-related ataxia (ATR) are two key kinases that are activated in extensive DNA damage [77]. When ATM and ATR are activated, selective autophagy of GATA4 is suppressed, resulting in an increase in GATA4 [75, 78]. In turn, GATA4 induces tumor necrosis factor receptor associated factor interacting protein 2 (TRAF3IP2) and interleukin 1A (IL1A), which activate NF-kB to initiate and maintain the SASP, thus facilitating senescence [75].

The Janus kinase/signal transducer and activator of transcription (JAK/STAT) pathway is one of the main mechanisms of cytokine regulation [79]. The JAK family consists of four members, including JAK1, JAK2, JAK3 and tyrosine kinase 2 (TYK2), and JAK1 and JAK2 play an important role in inflammatory signaling, regulation of growth hormones and other endocrine signals [79]. The transcription factor (TF) STAT plays a major role in regulating the expression of genes related to cell cycle progression, differentiation, proliferation and apoptosis [79]. Tumor necrosis factor-alpha (TNFα) is an important pro-inflammatory cytokine secreted by some types of senescent cells that can activate the JAK/STAT signaling pathway and persistent phosphorylation of STAT1/3 [80]. STAT1/3 activity is important for TNFα to induce SASP components in HUVECs, including IL8, IL6, IL1α, and IL1β [80].

The cyclic GMP-AMP synthase (cGAS)/stimulator of interferon genes (STING) pathway, which participates in innate immunity cytosolic DNA sensing, is involved in the regulation of the aging SASP and promotes senescence via paracrine signaling [81]. Abnormal cytoplasmic chromatin fragments (CCFs) are extruded from the nucleus of senescent cells [82]. Accumulating mitochondrial damage leads to an increase in reactive oxygen species (ROS) production, which activates the JNK kinase to regulate the formation of CCFs [82, 83]. CCFs trigger the SASP through the cGAS-STING pathway, which activates NF-κB [82, 83]. Activating the DNA-sensing cGAS/STING pathway can suppress EC proliferation and vascular repair [84].

Nrf2 is a basic leucine zipper protein (BZIP) and a major stress response factor that transcriptionally activates antioxidant and cytoprotective genes, regulates antioxidant protein expression, and protects cells from oxidative damage caused by injury and inflammation [85]. The degree of cellular senescence is negatively correlated with Nrf2 expression in vitro and in vivo[85]. When the nuclear factor Nrf2 is upregulated, ROS production decreases, DNA damage decreases, cell proliferation is promoted, and the SASP is reduced [85]. Inhibiting the key factor of Nrf2 mediates the premature aging phenotype, while activating Nrf2 can reduce the prevalence of atherosclerosis and stroke. Therefore, small molecule compounds that activate NRF2 are highly valuable [86].

The NAD^+^-dependent protein lysine deacylase of the Sirtuin family regulates various physiological functions, such as energy metabolism and the stress response[87]. The sirtuin subtype SIRT1-7 is considered an attractive target for the treatment of aging-related diseases[87]. Sirtuin1 (SIRT1) is an NAD-dependent class III histone deacetylase that not only deacetylates chromatin histones but also deacetylates various transcription factors, including STAT3, p53, forkhead transcription factor (FOXO) and NF-kB [88]. Das A *et al*. found that SIRT1 in ECs is a key mediator of the CM secretion of proangiogenic signals[89]. During aging, the level of NAD^+^ in ECs decreases, resulting in a decrease in SIRT1 activity and a decrease in the ability of endothelial cells to survive oxidative stress and respond to growth factor signals [89].

AMP-activated protein kinase (AMPK) is a key molecule in the regulation of biological energy metabolism, and the activation of AMPK can reduce oxidative stress, the activation of autophagy and the restoration of NAD+ synthesis [90, 91]. Cells lacking AMPK signaling cannot respond to physiological mitochondrial ROS signals, leading to disrupted mitochondrial ROS homeostasis and the induction of cellular senescence [92].

**Figure 3.**
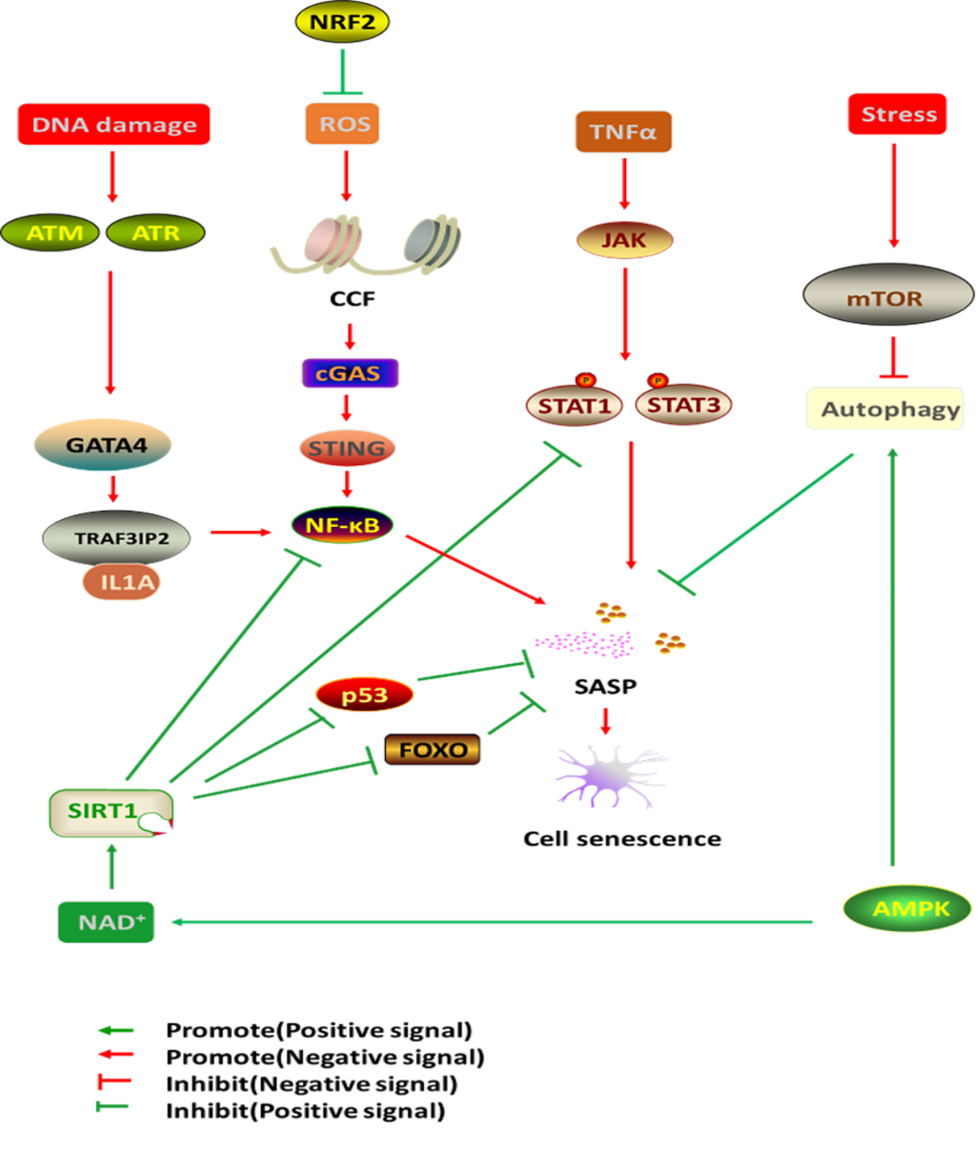
Pathways regulating cellular senescence and the SASP. The SASP is the most relevant phenotypic program in senescent cells. NF-κB is a central positive regulator of the SASP. On one hand, ROS and DAN damage can active cGAS/STING signal pathway and GATA4 signal pathway, thereby positively facilitating NF-κB and the SASP. On the other hand, SIRT1 serve as a negative regulator of the SASP. SIRT1 not only inhibit NF-κB but also suppress a series of transcription factors, such as STAT3, p53, forkhead transcription factor (FOXO). TNF-α can induce the SASP through activating JAK/STAT signal pathway. Autophagy inhibit the SASP, mTOR signal pathway represses autophagy. Oppositely, AMPK inhibit the SASP via promoting autophagy and activating SIRT1. Otherwise, NRF2 can repress the SASP by decreasing ROS.

A greater understanding of the mechanisms involved in senescence and SASP has highlighted several potential therapies for attenuating cellular senescence. The inhibition of MTOR and GATA4 or the activation of sirtuin and AMPK can appropriately enhance autophagy, reduce cell senescence and reduce SASP secretion. The activation of JAK/STAT and cGAS/STING could activate NF-κB to increase SASP. NRF2 decreases ROS production by regulating antioxidant protein expression, which can protect cells from oxidative damage and attenuate senescence (Fig. 3).

### Cellular senescence is regulated by noncoding RNAs

Noncoding RNAs (ncRNAs) account for nearly 98% of transcribed RNAs and lack the ability to be translated into proteins; ncRNAs include microRNAs (miRNAs), long noncoding RNAs (lncRNAs) and circular RNAs (circRNAs), which are ncRNAs that exist in the circulation[93]. NcRNAs are involved in various biological processes, such as regulating inflammation, cell proliferation, apoptosis, autophagy and senescence [94, 95] (Table 1).

MiRNAs are small single-stranded RNAs of approximately 19-24 nucleotides that regulate gene expression at the posttranscriptional level [96]. Mature miRNAs bind the complete complementary sites of the 3'-UTRs of the target mRNAs, reducing the stability of the target mRNA and inhibiting the protein translation of the target mRNA [97]. After hydrogen peroxide treatment, the expression of miR-217 in human umbilical vein ECs is increased, and miR-217 hinders the sirt1-induced regulation of NO synthesis by inhibiting the expression of its target gene sirt1, leading to the dysfunction of senescent ECs [98]. Sirt1 is also a target of miR-34a, which has been shown to induce senescence of VSMCs and CMs and increase myocardial fibrosis [99]. Sirt1 can be used as a treatment after AMI to downregulate miR-18a, which promotes the expression of brain-derived neurotrophic factor (BDNF), thereby inhibiting the AKT/mTOR pathway, promoting autophagy, and delaying senescence [100]. Delaying cellular senescence through miRNA is a feasible strategy for the treatment of cardiovascular diseases.

LncRNAs are endogenous single-stranded RNAs that are more than 200 bp long, lack the ability to encode proteins, and play an important role in cell proliferation, cell fate determination, apoptosis, and differentiation [101]. SENEBLOC, a long noncoding RNA, blocks cellular senescence by repressing the expression of p21 [102]. Rapamycin induces GUARDIN expression, and the lncRNA GUARDIN inhibits p21-dependent senescence by the LRP130-PGC1α-FOXO4 signaling axis [103]. S H *et al*. found that the lncRNA SNHG12 is a dynamic balance regulator of genome stability in atherosclerotic lesions [104]. Knocking out the lncRNA SNHG12 disrupts DNA damage repair, leading to vascular aging and accelerated atherosclerosis [105]. The lncRNA GAS5 can downregulate the expression of miR-223 through the PI3K/AKT signaling pathway to inhibit proliferation and promote senescence in EPCs [105].

**Table 1:** Non-coding RNAs that regulate cellular senescence.

Non-coding RNA	Effect on cellular senescence	Target gene	References
miRNA			
miR-217	ECs, positive	Sirt	[98]
miR-34a	VSMC and CM, positive	Sirt1	[99]
miR-18a	CMs, negative	BDNF, AKT/mTOR pathway	[100]
lncRNA			
SENEBLOC	ECs, negative	p21	[102]
GUARDIN	Non-small-cell lung carcinoma cells, HAFF, negative	LRP130/PGC1α/FOXO4 signaling axis	[103]
SNHG12	EC, positive		[104]
GAS5	EPC, positive	miR-223, PI3K/AKT pathway	[105]
CircRNA			
foxo3	Embryonic fibroblasts, positive	ID-1, E2F1, FAK and HIF1a	[110]
PVT1	Fibroblasts, negative	let-7, IGF2BP1, KRAS and HMGA2	[111]

CircRNAs constitute a group of endogenous noncoding RNAs with a covalently closed loop structure that lack a polyadenylated tail [106]. CircRNAs can act as miRNA sponges to prevent interaction between miRNAs and mRNAs, indirectly regulating the expression of downstream miRNA target genes or affecting gene expression by regulating splicing, transcription and interaction with RNA-binding proteins [107]. CircRNAs have been shown to be good therapeutic targets and diagnostic biomarkers of many diseases and regulate cell proliferation by affecting signaling pathways, transcription factors and cell cycle checkpoint regulators; in addition, circRNAs may be involved in the regulation of aging and aging-related diseases [107-109]. circ-foxo3 interacts with the antiaging protein ID-1, transcription factor E2F1, and the antistress proteins FAK and HIF1α [110]. The ectopic expression of CIRC-Foxo3 promotes cellular senescence, while silencing CIRC-Foxo3 reduces cellular senescence and apoptosis [110]. let-7 increases cellular senescence and inhibits the production of antiaging and proliferation-promoting proteins, which has been shown to promote senescence [111]. Inhibiting the level of CircPVT1 can inhibit let-7, reverse several target genes of let-7 that encode proliferation proteins, and inhibit senescence [111].

## Cellular senescence affects cardiac regeneration and repair

The proliferation, migration, differentiation, homing and paracrine abilities of senescent cells are impaired, which impairs the function of angiogenesis and myogenesis after MI, leading to ventricular remodeling and heart function deterioration, ultimately causing heart failure [29, 112-115].

### ECs

ECs form the inner surface of blood vessels and play an important role in vascular biology, including vasodilation, hormone transport and angiogenesis [116]. Hypercholesterolemia, diabetes and metabolic syndrome induce oxidative stress generated by mitochondrial dysfunction, accelerating the senescent process of ECs by initiating telomere shortening [117, 118]. Senescent ECs induce endothelial dysfunction, which can also lead to vasoconstriction and a predisposition to angina pectoris and ischemic heart injury [119]. In addition, senescent ECs release endothelial-derived microparticles, increase the expression and activity of tissue factors and increase the aggregation of platelets, resulting in thrombosis [120]. Senescent ECs affect cellular proliferation and angiogenesis [121], impairing repair after MI. Chi C et al found that activating the antiaging protein Nrf2/HO-1 prevents human endothelial cellular senescence and improves the pathological changes in cardiovascular diseases, such as thrombosis, MI, and atherosclerosis [122].

### CMs

CMs belong to postmitotic cells, and biomechanical stress, oxidative stress, and inflammation can also cause telomere attrition, eventually leading to cellular senescence [123, 124]. Hyperglycemia accelerates senescence in CMs by inducing various cellular stressors, such as oxidative stress, DNA damage, and telomere erosion, which is a well-accepted reason for the increased incidence of cardiovascular disease in diabetic individuals [118]. Ischemic injury caused by MI initiates DNA damage, oxidative stress and mitochondrial dysfunction, which are reasons for CM senescence [125]. Many CMs are lost after myocardial ischemic injury, resulting in the replacement of cardiac tissue with a nonfunctional extracellular matrix network structure, forming scars and limiting cardiac output and cardiac function [126]. Therefore, delaying the senescence of CMs or promoting the proliferation of existing CMs is particularly important for improving cardiac function. Heme oxygenase-1 (HO-1) plays a role in reducing cellular senescence in the process of MI and cardiac aging [125]. Ebeid DE et al found that PIM1 is a myocardial protective kinase that delays cellular senescence by maintaining the telomere length [123]. Rb1 and Meis homeobox 2 (Meis2) is a set of cell cycle inhibitors [126]. Alam P *et al*. found that terminally differentiated CMs can re-enter the cell cycle by inhibiting Rb1 and Meis2 in vitro and in vivo, thereby improving angiogenesis and heart repair after acute ischemic injury [126].

### VSMCs

As a basic component of the vascular wall and the only cell type in the middle layer of the artery, VSMCs play an important role in vascular physiological function [127]. VSMC senescence may be triggered by many factors, such as angiotensin II, oxidative stress, inflammation, DNA damage, and small molecule compounds [127]. Senescent VSMCs appear in atherosclerotic intima and are related to plaque stability; plaque instability is an important cause of plaque rupture [127]. Therefore, delaying senescent VSMCs can stabilize atherosclerotic plaques and reduce the incidence of adverse events in vascular diseases, including MI. Doxorubicin induces VSMC senescence by increasing mTOR activity and decreasing the autophagy protein levels, while the inhibition of the mTOR pathway leads to a sharp reduction in the number senescent cells [128]. Rapamycin can activate autophagy to inhibit senescence in VSMCs [73]. VSMC calcification is also a manifestation of cellular senescence and widely exists in various cardiovascular diseases [129]. Bartoli-Leonard F et al found that downregulating the expression of SIRT1 promotes calcification in rat VSMCs cultured in vitro; thus, targeting SIRT1 can regulate cell calcification and senescence [130].

### EPCs

EPCs can not only promote the recovery of ECs after injury but also stimulate angiogenesis after AMI[131]. EPCs can be significantly mobilized from the bone marrow to the peripheral circulation, home to the ischemic heart, differentiate into mature ECs, and initiate the repair process of neovascularization [132, 133]. Long-term exposure to risk factors, such as smoking, glycosuria, hypertension, hyperlipidemia and/or cardiovascular disease, in aging can reduce the number of EPCs, and a low EPC count is associated with a high mortality in patients with coronary heart disease [134]. Since maintaining the number of EPCs is highly important, some methods that attenuate cellular senescence and maintain the ability of proliferation should be considered. The expression of NRF2 and its target genes also declines with aging, and R W et al found that the downregulation of Nrf2 expression significantly impacted EPC survival and function and induced senescence [131]. These authors also found that the upregulation of NRF2 can suppress oxidative stress in EPCs during aging and restore the function of aged EPCs [131]. Song X et al proved that the combined stimulation of high glucose and free fatty acids can accelerate the senescence of EPCs and inhibit EPC reendothelialization, which is regulated by the PGC-1α/SIRT1 signaling pathway [135]. Lin *et al*. showed that rhynchophylline attenuates the senescence of EPCs by activating the AMPK signaling pathway, which enhances autophagy [136].

### MSCs

It is generally believed that MSCs can produce various nutritional factors and immunomodulatory factors after MI, repair or replace original damaged CMs, recruit resident heart ECs to the infarct marginal zone, promote angiogenesis, improve the myocardial blood supply, recover myocardial function, and delay ventricular remodeling [137-139]. MSCs from older and younger patients were transplanted into a rat MI model, and the results showed that the mRNA and protein expression levels of VEGF and Bcl-2 from the younger patients were higher than those from the older patients and that the MSCs from the younger patients exhibited reduced CM apoptosis and improved myocardial contractility[140]. Under the conditions of serum deprivation, hypoxia and oxidative stress, proliferation and paracrine effects are impaired, inducing senescence or apoptosis, and, therefore, limit the cardioprotective effects after MI [141]. The overexpression of ERBB4 in MSCs from older patients promotes angiogenesis, the MI size and improves cardiac function after MI by activating PI3K/AKT and MAPK/ERK [142]. Liu X et al found that the therapeutic effect of senescent MSCs on MI is worse than that of normal MSCs, but inhibiting the expression of miR-206 in senescent MSCs can improve the therapeutic effect, reduce the area of MI and increase angiogenesis [141]. After the transplantation of MSCs overexpressing Sirtuin3, the infarct size was reduced, which improved the efficacy of the MSC transplantation in the treatment of ischemic hearts in older patients [143].

**Figure 4.**
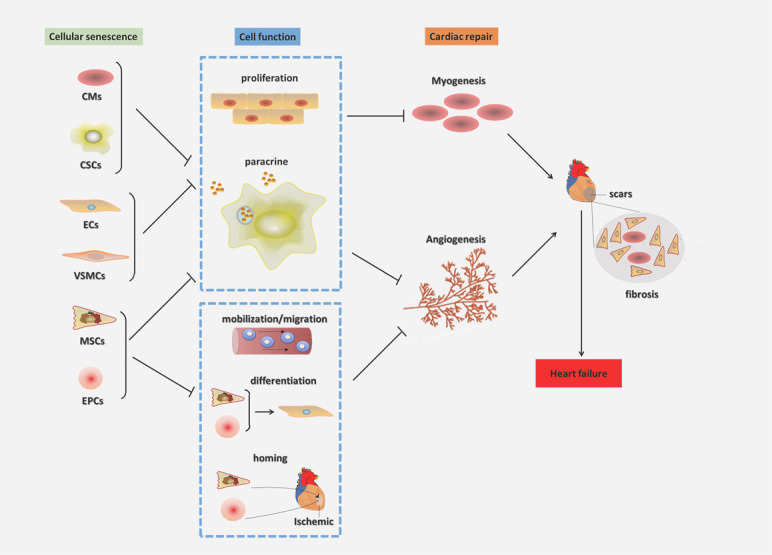
Cellular senescence affects cardiac regeneration and repair. The proliferation and paracrine of senescent CMs CPCs ECs VSMCs MSCs EPCs were weaken. The decrease of CMs and CPCs attenuate myogenesis after MI. The myocardial cells in the infarct area are replaced by fibroblasts, and cardiac scars are formed. The abilities of senescent MSCs and EPCs to mobilize, migrate, differentiate, and home to the ischemic area of the heart decrease, resulting in the reduction of ECs and VSCs, which inhibit angiogenesis and cardiac repair. The senescent cells hinder angiogenesis and myogenesis after MI, leading to ventricular remodeling and heart function deteriorating, finally causing heart failure.

### CSCs

With aging, the accumulation and activation of aging factors cause DNA damageand changes in the telomere-telomerase system, resulting in CSC senescence and the potential to lose or decrease the number of CSCs [144]. Senescent CSCs show a weakened potential for self-renewal, differentiation and regeneration and have a negative effect on surrounding CSCs, accelerating their senescence [18]. c-kit^+^ CSCs have the potential to differentiate into CMs and improve cardiac function after MI *in vivo* [145]. However, in vitro and in vivo, only less than 1% of myocardial c-kit^+^ CSCs have the potential for colony formation, self-renewal and cardiac multilineage differentiation [146]. Whether CSCs can differentiate into CMs remains controversial, but it is certain that both the CSC number and paracrine effects are beneficial for delaying ventricular remodeling after MI. CSCs that secrete cytokines and growth factors can exert paracrine activity on endogenous CSCs, allowing them to proliferate and differentiate into adult CMs [114]. These senescent CSCs can reverse senescence by in vitro genetic modifications, regulate aging-related pathways by administration, and optimize extracellular factors to restore stem cell viability [147]. The transplantation of CPC into an infarcted myocardium can improve myocardial contractility and ventricular remodeling, inhibit inflammation, and increase CM survival, myogenesis and angiogenesis/neovascularization. Saheera S *et al*. found that oxidative stress and senescence of CSCs were alleviated after treatment with metoprolol, which increased the migration and proliferation of CSCs and was beneficial in delaying progressive cardiac remodeling [148]. Insulin-like growth factor-1 is a synthetic growth hormone that regulates cell proliferation, differentiation, senescence and death; Sun Y *et al*. found that IGF-1 promotes the proliferation and migration of CSCs by activating the PI3K/AKT/DNMT signaling pathway [149].

Senescent cardiovascular cells contribute to cardiovascular disease development and weaken the repair of cardiac damage. Aging, hypercholesterolemia, diabetes, metabolic syndrome, ischemic injury and various other internal and external factors induce oxidative stress, DNA damage, and telomere erosion, thereby accelerating cellular senescence. Senescence of ECs, VSMCs, EPCs and MSCs impairs the cardiac vasculature and reduce angiogenesis, while senescent CMs and CSCs mainly affect myogenesis and myocardial contractility. Improving cardiac function to target senescence by treatment strategies is essential (Fig. 4).

## Treatment strategies targeting senescence

Since senescent cells are potentially involved in cardiac vascular disease processes, avoiding the risk factors that induce cellular senescence, eliminating senescent cells and attenuating the SASP have emerged as attractive therapeutic strategies (Table 2).

**Table 2:** Senescent cell-related molecules, signaling pathways, regulation and intervention.

Molecules/ signaling pathways	Senescence	Regulation	Related senescent cells	Pharmacological intervention
mTOR	Promote	SASP [73] Autophagy [72]	VSMCs [72]	Rapamycin [164]
GATA4	Promote	SASP [75,78]	MSCs [76]	Flavonoid4,4′dimethoxychalcone [170]
JAK/STAT	Promote	SASP [80]	HUVECs [80]	Roxolitinib [171]
cGAS/STING	Promote	SASP [82,83]	ECs[84]	Inhibition of STING [172]
NRF2	Inhibit	ROS [85] SASP [85]	EPCs [131]	Resveratrol [85]
Sirtuin	Inhibit	Autophagy [88] ROS [88]	VSMCs[162]	Resveratrol [162] nicotinamide mononucleotide [89]
AMPK	Inhibit Inhibit[165-168]	ROS [90] Autophagy [90] Apoptosis[165]	EPCs[134] ECs[166] MSCs[166]	Metformin [164] Senolytic drugs[165]

### Avoiding the risk factors that induce cellular senescence

There are many risk factors for cellular senescence, which can be prevented by fully avoiding the risk factors and delaying the occurrence of cardiovascular diseases. Conley SM *et al*. found that obesity changes the function and characteristics of adipose tissue-derived MSCs and induces their senescence, injuring the angiogenic potential of MSCs [150]. Calorie restriction is currently the most effective nongenetic intervention to delay the senescent phenotype [151]. FontanaL et al showed that calorie restriction can prevent the accumulation of senescent cells in mice and humans and prevent the potential side effects of aging [151]. Zhang JJ *et al*. established a hyperlipidemia model by feeding rats a high-fat diet, leading to EC senescence by elevating the blood lipid levels [152]. However, atorvastatin k can inhibit EC senescence by regulating the blood lipid levels [153]. Smoking induces DNA damage in a manner similar to obesity, which can aggravate senescence and trigger inflammation through the cGAS/STING pathway [154], and a sedentary lifestyle is associated with increased inflammation and oxidative stress [155]. In a high-glucose microenvironment, circulating EPCs, ECs and VSMCs undergo a decrease in telomerase activity and cellular senescence, and the control of blood glucose is an important strategy for controlling the risk factors of cellular senescence [135, 156]. Insufficient sleep can lead to shortened telomeres and a significant increase in specific DNA damage, the accumulation of free radicals and the production of SASP factors in mice [157]. The working environment, stress and duration can also accelerate telomere depletion; for example, Ridout KK *et al*. found that the stress that occurs during the training of physicians can lead to accelerated cellular senescence [158]. Exercise has the potential to counteract many pathological processes considered to lead to age-related heart failure, including senescence, inflammation, mitochondrial dysfunction and a decrease in CMs [155, 159]. The catabolism of skeletal muscle activated by aerobic exercise may prevent cellular senescence partially by regulating the cell cycle [155, 159]. Improving a poor living environment and lifestyle, such as a high-fat diet, a high-glucose diet, smoking, sedentary habits, and sleep deprivation, is highly important for preventing the occurrence of cellular aging and aging-related cardiovascular diseases.

### Targeting the mechanisms that regulate senescent cells

Targeting the metabolic pathways on which senescent cells depend has been studied to prevent, mitigate, and reverse cardiovascular disease related to cellular senescence. Resveratrol and melatonin interventions with antioxidant and anti-inflammatory effects can be used to prevent/delay senescence [160]. Preclinical studies have shown that pretreatment with resveratrol before myocardial ischemia can decrease the infarct size and reducing arrhythmia [161]. Resveratrol is one of the most powerful activators of SIRT1 and NRF2,which delay cellular senescence and decrease the SASP [131, 162]. In addition, melatonin plays an important role and exerts an antioxidant effect, and daily melatonin treatment protected the vascular endothelium against aging-, oxidative stress-, and ischemia-induced damage likely by upregulating the SIRT signaling pathway [163]. Besides, enhancing autophagy is significant for delaying senescence. The inhibition of the MTOR pathway by rapamycin or activation of the AMPK pathway by metformin can promote autophagy, thereby delaying cellular senescence [164]..

### Eliminating senescent cells

Senolytic drugs are agents that selectively induce apoptosis in senescent cells, including small molecules, peptides, and antibodies [165]. The first senolytic agent was reported in 2015, and that article showed that dasatinib eliminated senescent human fat cell progenitors, while quercetin was more effective against senescent human endothelial cells and mouse BM-MSCs [166]. It has been shown that transplanting relatively small numbers of senescent cells into young mice is sufficient to cause persistent physical dysfunction, while dasatinib plus quercetin selectively eliminated senescent cells, alleviated physical dysfunction and increased post-treatment survival by 36% while reducing the mortality hazard to 65%[167]. Since the first human trial of senolytics indicated potential for clinically meaningful improvements, more clinical trials of senolytics are under way or will begin soon[168]. Senolytics is promising for eliminating senescent cells and improving cellular senescence-related diseases.

### Targeting the SASP pathway

Senescent cells secrete the SASP factors, including IL-6, IL-1α, IL-1β and TNF-α; affect neighboring cells; destroy the stem cell niche; change the extracellular matrix; and induce secondary senescence. Rapamycin inhibits the SASP by downregulating IL-6, IL-8, monocytes, chemotactic proteins, etc. [74, 169]. Flavonoid 4,4′dimethoxychalcone (DMC) is considered an antiaging compound that has regulate the SASPs effects in mice and promotes autophagy through a pathway that involves specific GATA transcription factors [170]. Roxolitinib is a selective JAK1/2 inhibitor with a minimal effect on the generation and function of immune cells, reducing the inflammation of premature senescent cells and improving cell homeostasis [171]. Recently, a specific covalent inhibitor of STING showed a very high therapeutic effect in inflammatory diseases because it does not inhibit other innate immune pathways [172]. Jesús G *et al*. reported that the RNA-binding protein ZFP36L1 is a direct regulator of the SASP that can degrade the transcripts of numerous SASP components and rescue growth arrest [173].

### Current limitations of targeting cellular senescence

Drug scavenging of aging cells in aging mice reduced age-dependent myocardial remodeling, weakened the expression of fibrogenic mediators, promoted cardiac function after MI, and finally improved the survival rate [18]. However, a limitation of this method is that it cannot distinguish the senescent cells that are truly working, and the removal of senescent cells in nonheart organs may affect survival after MI [18]. Therefore, it is still necessary to further study methods to specifically remove senescent cells and improve diseases. Senescent cells and the SASP factors can promote aging-related diseases and cancer; however, delaying cellular senescence or eliminating senescent cells is not always beneficial for the body. Cellular senescence can prevent the occurrence and development of tumors; however, senescent cells and the SASP may also provide an important local but persistent oncogenic stress signal, thus promoting immune surveillance of cancer cells [64]. Therefore, how to balance the role of tumor and antitumor promotion in the treatment of cellular senescence is a problem that needs to be explored. Another potential problem is that targeting the SASP may impair other physiological effects in addition to the proinflammatory effect of senescence. For example, NF-κB plays an important role in the control of acute inflammation and the immune response, while mTOR regulates cell growth, proliferation, protein synthesis and self-regulation [174]. Therefore, treatment strategies should consider the SASP inhibition without affecting other functions.

## Conclusion

With aging and changes in the body's microenvironment, senescent cell accumulation increases and is closely related to cardiovascular diseases. On the one hand, cellular senescence promotes vascular aging and atherosclerosis, accelerating the process of IHD. On the other hand, cellular senescence has adverse effects on angiogenesis and myogenesis, impairing regeneration and repair after MI and leading to a deterioration of cardiac function. In recent years, significant progress has been achieved in studying the mechanisms of cellular senescence and the SASP. Avoiding the risk factors of cellular senescence can prevent cardiovascular disease; targeting and attenuating cellular senescence or clearing senescent cells repair damage after MI; and targeting the regulation of the SASP can reduce the negative effects of inflammation. However, there are some problems that urgently need to be solved in future research and clinical trials. How can senescent cells be precisely and specifically targeted? How can the protumoral and antitumoral roles in the treatment of cellular senescence be balanced? How can the SASP be suppressed without affecting other functions, such as immune surveillance? The targeted regulation of cellular senescence is required to not only control the mechanism of senescence but also repair DNA damage and reduce the occurrence of cellular senescence, apoptosis and tumors; this regulation will be an important aspect of precision medicine.
